# Strengthening adolescents’ critical health literacy and scientific literacy to tackle mis- and dis-information. A feasibility study in Switzerland

**DOI:** 10.3389/fpubh.2023.1183838

**Published:** 2023-09-01

**Authors:** Maddalena Fiordelli, Nicola Diviani, Ramona Farina, Paolo Pellicini, Alberto Ghirimoldi, Sara Rubinelli

**Affiliations:** ^1^Institute of Public Health, Università della Svizzera italiana, Lugano, Switzerland; ^2^Faculty of Health Sciences and Medicine, University of Lucerne, Lucerne, Switzerland; ^3^Swiss Paraplegic Research, Nottwil, Switzerland; ^4^Università della Svizzera italiana, Lugano, Switzerland; ^5^Istituto Elvetico Salesiani Don Bosco, Lugano, Switzerland

**Keywords:** infodemic, critical health literacy, argumentation skills, scientific literacy, critical thinking

## Abstract

**Objectives:**

We aimed to develop and test the feasibility of a critical health literacy (CHL) and science literacy (SL) training course targeting secondary school students in Switzerland.

**Methods:**

Using a community-based participatory approach, we developed a two-block training program, the first centered on argumentation skills and the second on scientific skills. We combined an ex-cathedra and a flipped-classroom approach, providing students with a deep understanding of CHL and SL concepts and the translational capability of implementing theoretical notions to real case scenarios. The feasibility study was designed as a one-group pretest-posttest quasi-experiment. Beyond socio-demographics, questionnaires included measures of CHL, SL, trust in science, and perceived quality of the course.

**Results:**

The curriculum was feasible and well-accepted by the target groups, teachers, and students. Students convincingly specified their perceived personal benefits associated with a positive change in CHL and SL scores after the training course.

**Conclusion:**

Training CHL and SL in secondary school students is feasible and can improve their competencies. Results from present study can inform a large-scale study.

## 1. Introduction

The global spread of the COVID-19 disease has been accompanied by what the World Health Organization (WHO) defines as an *infodemic*. The term refers to an overabundance of information (real or not) whose growth can occur exponentially in a short time (1). Among low-quality information spread through an infodemic, we may distinguish between mis- and disinformation. The former refers to false or misleading information disseminated by a source that believes in its truth. In contrast, the latter refers to false or misleading information shared by a person knowing it is false (2, 3). An infodemic can harm to health because it generates confusion, favors risk-taking behaviors, leads to mistrust in health authorities, and exacerbates or extends outbreaks when people are uncertain about health-protective behavior. Adverse consequences arising from infodemics have been extensively documented, spanning from individual repercussions to those affecting health systems and communities at large (4). Infodemics around the globe appeared as a vast phenomenon, especially during the COVID-19 pandemic, because of digitization, through which information can spread at an unprecedented speed (5). This may help fill information voids, but it can amplify dangerous messages (1). In this landscape, reflections on the skills individuals need to evaluate health information correctly and how to enhance them are of fundamental importance (4, 6).

The challenges posed by infodemics also highlight issues that have been addressed in the field of public understanding of science for nearly 30 years (7). There is a growing need to promote scientific comprehension and employ a scientific approach to science communication, fostering a cultural shift. This becomes particularly urgent in the context of democracy (8). In the sphere of health literacy and health information appraisal, there is evidence of the need to empower people to develop skills in critical health literacy (CHL) and scientific literacy (SL) (8–10). CHL is a complex construct encompassing both knowledge, personal skills, social determinants, and engagement in collective actions (11). This construct covers skills and dispositions that equip people to engage in decision-making based on reasons and values as independent thinkers (11). SL refers to an individual’s understanding of scientific concepts, phenomena, and processes and their ability to apply this knowledge to new and, at times, non-scientific situations (12).

Both CHL and SL are among the “21st-century skills” and a crucial goal of education (13). Within a democratic society, they are essential for individuals to become active citizens. This is even more true in the context highlighted at this section’s beginning, where infodemics pose many complex challenges in health information appraisal. However, despite their proclaimed relevance, there is scant evidence on the skills these two constructs effectively involve and how to promote them (14). To address this gap, we designed an intervention where we operationalized some of the skills of CHL, with a specific focus on information appraisal, and SL and evaluated its feasibility and acceptability in a cohort of adolescents. For CHL we focused on information appraisal skills, and specifically on argumentation skills, i.e., those skills needed to evaluate whether an argument is sound, valid, appropriate, or fallacious when it is persuasive and of high quality or bad quality and manipulative (15). Enhancing argumentation skills can support in recognition of argumentative health information, in the identification of supporting reasons for claims, and in the development of critical questions (16). Hence, it is imperative to equip citizens with these skills to effectively combat the proliferation of misinformation. Science employs various methodologies to reach its results, starting from the study of a phenomenon, obtaining reliable results, and ultimately publishing these findings in peer-reviewed journals. The OECD PISA Framework described SL as “the ability to engage with science-related issues, and with the ideas of science, as a reflective citizen” (17). SL equips citizens with the ability to engage critically with scientific information, reinforcing their rational thinking. These skills are crucial to avoiding a decision-making process solely based on personal opinions, experiences, or beliefs (18). Our emphasis was directed toward SL skills in terms of the capability to comprehend the evolution of scientific discussions and the scientific methodology, rather than encompassing a wider disposition toward science (19, 20).

We targeted adolescents, as there is much evidence that thinking skills are best developed early in life. Adolescence is the main phase of human development characterized by many cognitive, emotional, and physical changes. According to Piaget, adolescents are empowering cognitive abilities, developing an improved capacity for processing information, thinking more about abstract concepts, scientific reasoning, hypothesis testing, and using reasoning skills aside from achievement of greater autonomy (21, 22). These changes make adolescence a reasonable period to introduce CHL and SL interventions because of a potential direct impact on adulthood (23, 24).

We developed a CHL and SL training course and pilot-tested it with teachers and students in a central secondary school in southern Switzerland (Ticino). Our aim was to test the acceptability and the feasibility of the training.

## 2. Methods

The feasibility study was a one-group pretest-posttest quasi-experimental design. Students participating in the training filled in a questionnaire at the beginning and the end of the training.

### 2.1. Design principles of the CHL and SL course for secondary school students

#### 2.1.1. Course content and pedagogical approach

We concentrated on topics that are recognized in the literature for their significance in information evaluation to determine the content of the training course. We aimed to increase adolescents’, awareness of when and how they are in front of argumentative content, providing them with instruments to make better choices when engaging with health information and enhancing the ability to recognize if the information is scientific and grounded on evidence. The course was structured into two blocks: the first centered on argumentation skills and the second on scientific skills (Table 1). The blocks combined ex-cathedra and flipped classroom approaches (25), using a learner-centered approach, and providing students with a deep understanding of CHL and SL concepts and the translational capability of implementing theoretical notions to real case scenarios.

**Table 1:** Course contents.

Critical health literacy (4 h)	Scientific literacy (4 h)
Infodemics and the importance of critical thinking The elaboration of health information The evaluation of the source’s credibility Persuasion in the online world	What science is and how it gets to its results From study-design to publication Pseudoscience

### 2.2. Theoretical frameworks

#### 2.2.1. Critical health literacy

Critical skills in argumentation are described in the field of argumentation theory, which studies how to support claims by reasons and their soundness. As defined (16, 26, p. 1), argumentation is “a verbal, social, and rational activity aimed at convincing a reasonable critic of the acceptability of a standpoint by putting forward a constellation of propositions justifying or refuting the proposition expressed in the standpoint.” Argumentation theory guides people to understand whether claims are soundly supported and teaches how to determine whether and why arguments are mistaken, unrelated, or manipulative.

We specify below the content and theoretical frameworks of each module of the first block.

1) Infodemics and the importance of critical thinking.

Module 1 defined the concepts of disinformation, misinformation, and malinformation. It also focused on those affective and cognitive elements that lead people to form dis-informed views. Critical thinking and its characteristics were introduced with a focus on deductive and inductive reasoning and argumentation and its structure by identifying basic argumentation typologies. Fallacies were described as a case of unsound argumentation, with different forms and typologies, often conducting to flaws in reasoning.

2) The elaboration of health information.

Using the “nudge” approach (27), together with the Elaboration Likelihood Model (28), module 2 explained the differences between the slower thinking process (rationale system) and the instantaneous one (impulsive system), as well between the central vs. the peripheral thinking pathway. Particular emphasis was on heuristics, defined as shortcuts that may lead to inaccurate judgment (bias) (14). Students were invited to reflect on the benefits and drawbacks of heuristics and how they work, helping them make more accurate decisions. Students learned to differentiate stereotypes, prejudices, and ideologies, defined as the main barriers to critical thinking, and to distinguish opinions and facts in a piece of information.

3) The evaluation of the source’s credibility.

Module 3 was devoted to distinguishing between reliable and unreliable sources using specific criteria well-developed in argumentation theory. The module provided tools to evaluate information reliability on the Internet since this is known to be the main challenge (29). The focus was on topics such as the competence, reliability, and attractiveness of sources (30), with a spotlight on competence as an essential characteristic of credibility when people make claims about science and its results.

4) Persuasion in the online world.

The last module addressed mis/disinformation online with a focus on influencers. Influencers who have many followers are good communicators. Yet, while trendy influencers promote mis/disinformation and make it appealing and persuasive, scientific influencers use their skills and expertise to explain complex scientific concepts, debunk online mis- and disinformation, and guide followers’ decision-making (31).

#### 2.2.2. Scientific literacy

Our course focuses on essential characteristics of science, from study design and methods to publication, that can guide students in evaluating whether claims are scientific. The module discussed the issue of trust in science as the source of evidence-based knowledge that can orient human behavior. Trust in science can be defined as “a perception of scientists as credible, likely to tell the truth and share the public’s interest” (32). But, as remarkably addressed by Nichols (33), this society often contrasts the value of expertise and the scientific approach, promoting a more relativistic view that does not differentiate between the experience of an individual or more individuals and what can be generalized.

We specify below the content and theoretical frameworks of each module of the scientific literacy block.

1) What science is and how it gets to its results.

The definition of science was introduced using the definition given by Rubinelli et al. (34). The differences between “soft” and “hard” and between “formal,” “empiric,” and “applied” science were explored through some exercises. The main emphasis was placed on the scientific method, the various stages of the process, distinguishing between qualitative and quantitative methods, and describing the levels of the scientific evidence pyramid.

2) From study design to publication

Module 2 focused on the scientific discourse and the different dissemination outlets. It covered the publication of study results through the peer-review process and the existence of metrics for evaluating the cumulative impact of an author’s scholarly output.

3) Pseudoscience

The last module explained how to discriminate science from pseudoscience and the consequences for individuals and societies when pseudoscience spreads. Participants were instructed to identify conspiracy theories and the psychological factors driving their popularity.

### 2.3. Course format

The course was structured in two 4-h morning sessions (one per block), which consisted of frontal lectures with practical exercises and questions between the modules to promote interactivity. Each session encompassed real-life cases, and the students applied the acquired skills to scientific questions, activities, standpoints, and controversies. At the end of the first block, four written posts containing false information were presented to the students divided into groups. Each group examined and discussed their post using critical thinking and notions learned during the course and presented in the plenary. At the end of the second block, the class watched and discussed two ironic cartoon videos deconstructing conspiracy theories.

### 2.4. Research approach and selection of participants

The courses were arranged from mid-January to mid-February and held by the authors of this paper. This study builds on the community-based participatory research approach (35), in collaboration with three teachers throughout the research process. We approached these teachers because they were designated by the school manager, our very first contact point. We structured meetings to validate the contents and methods of the course. Lessons were held in the institute thanks to the cooperation of the lecturer of human sciences (PP), history, and philosophy contacted by the PIs in October 2021. An initial meeting took place in October 2021 to introduce key elements of the project and its purpose to the project team and the teachers. In December 2021, a second meeting was held to discuss the objectives and define the curriculum contents and the schedule. The choice of targeting students from last year high school classes (scientific, linguistic, and human sciences) and the second-to-last year class of the scientific major was made during these meetings. At the end of the training course, in April 2022, the study’s results were presented in a meeting where the teachers provided further feedback.

### 2.5. Data collection

Data were collected through two online questionnaires to the students at the beginning of the first class and at the end of the second meeting. The variables collected are specified below.

#### 2.5.1. Socio-demographic data and individual inclination in critical thinking

The first questionnaire collected socio-demographic characteristics (age, gender, and nationality) and a self-assessment of individual inclination toward critical thinking. The final questionnaire was composed of 11 items assessing the disposition of the individual to reasoning, arguing, and making decisions, adapted from the Critical Thinking Disposition Scale (36). The first seven items evaluated Critical Openness, i.e., the disposition to be open to new ideas, critical in evaluating these ideas and modifying one’s thinking by considering convincing evidence. The last ones assessed Reflective Skepticism, i.e., the tendency to learn from one’s past experiences and be questioning evidence (36). Students were asked to rate their level of agreement with statements on a scale from 1 (totally disagree) to 5 (totally agree).

#### 2.5.2. Critical health literacy

The scale included ten items exploring the perceived ability of students to refrain from drawing immediate conclusions in front of news arousing emotions, to verify the credibility of a source, to assess the value of an opinion, and to recognize bias and fake news (to consult the scale see Supplementary material). The scale was developed among the project team, and face validity was discussed, then it was pilot tested with a group of university students, Students were asked to rate their level of agreement with statements on a scale from 1 (totally disagree) to 5 (totally agree). The scale, designed by the authors of this paper, was part of both questionnaires. Reliability score was 0.57 (Chronbach’s alpha).

#### 2.5.3. Scientific literacy

The 10 items of the survey examined students’ perceived ability to define scientific theories’ main characteristics, differentiate between conspiracy theories and scientific ones, and between scientific evidence and opinions, ideologies, and superstitions, and describe the processes in the scientific method. The scale was developed among the project team, and face validity was discussed, then it was pilot tested with a group of university students. Students were asked to rate their level of agreement with statements on a scale from 1 (totally disagree) to 5 (totally agree). The scale was part of both questionnaires (to consult the scale see Supplementary material). Reliability score of the scale was 0.70 (Chronbach’s alpha).

#### 2.5.4 Trust in science

The questionnaire was composed of (1) three items to evaluate the distrust in science and (2) two items measuring the trust in science. Specifically, the scale included the item highlighting the lack of trust in scientists, scientific theories, and more general scientific society. The other two items evaluated the trust in scientists and science. The items were an Italian adaptation of the scale developed by Sulik and colleagues in 2021 to evaluate the relationship between trust in science and the acceptance of COVID-19 protective measures (37) and the questionnaire Trust in Science and Scientists Inventory (TSSI) (38) to measure generic trust in scientists and science (39). Students were asked to rate their level of agreement with statements on a scale from 1 (totally disagree) to 5 (totally agree). The scale was part of both questionnaires. Reliability score of the scale was 0.56 (Chronbach’s alpha).

#### 2.5.5. Perceived quality of the course

At the end of the training, students evaluated the course. They assessed the relevance of the training in terms of clarity, satisfaction, applicability to daily life issues, and interest. The individual perceived quality was evaluated using a 5-point Likert scale ranging from 1 = strongly disagree to 5 = strongly agree. A qualitative evaluation of the course to gain in-depth insight into its perceived value followed through three open-ended questions. We analyzed thematically the three open answers.

#### 2.5.6. Statistical analysis

Analysis was carried out using IBM SPSS version 26. Descriptive statistics were computed for different sample characteristics and CHL and SL scores. Data were distributed normally, and we compared before and after scores with paired *T*-tests. Pearson’s correlation coefficient was calculated to find correlations between different scores. One-way ANOVA was employed to compare groups across multiple specializations. The significance level for all the statistical tests was kept at *p* < 0.05.

## 3. Results

Characteristics of the sample are shown in Table 2. In total, 97 students were exposed to the intervention. Seventy-two students (74% response rate) completed both questionnaires. Four classes attended the courses: three from the 4th year and the other from the 3rd year. The mean age was 18 years old. Students attended human science (40%), scientific (32%), and scientific/linguistic high school (28%). Most participants were from Switzerland (51%) and Italy (44%), and the remaining students were from other countries (5%).

**Table 2:** Participants characteristics.

Variables	*N* = 72
**Age**	
Min	16
Max	21
Mean (SD)	18 (0.934)
**Gender**	
Male	53%
Female	46%
Other	1%
**Grade**	
3rd	25%
4th	75%
**School major**	
Human science	40%
Scientific	32%
Linguistic and scientific	28%
**Nationality**	
Switzerland	51%
Italy	44%
Other	5%

### 3.1. Individual inclination in critical thinking before the training course

As regards disposition toward critical thinking, on average, the students scored 4.01 on a scale from 1 (totally disagree) to 5 (totally agree). As reported in Table 3, the highest mean score of the questionnaire was 4.39 for item 6 (“It’s important to understand other people’s viewpoint on an issue”), and the lowest was 3.76 for item 2 (“I often use new ideas to shape (modify) the way I do things”).

**Table 3:** Participants’ assessment of the critical thinking inclination.

Item	Mean (SD)
I usually try to think about the bigger picture during a discussion	4.01 (0.646)
I often use new ideas to shape (modify) the way I do things	3.76 (0.903)
I use more than one source to find out information for myself	4.37 (0.663)
I am often on the lookout for new ideas	3.97 (0.826)
I sometimes find a good argument that challenges some of my firmly held beliefs	3.81 (0.858)
It is important to understand other people viewpoint on an issue	4.39 (0.771)
It is important to justify the choices I make	3.83 (1.167)
I often re-evaluate my experiences so that I can learn from them	3.91 (0.697)
I usually check the credibility of the source of information before making judgments	3.97 (0.900)
I usually think about the wider implications of a decision before taking action	4.06 (0.817)
I often think about my actions to see whether I could improve them	4.02 (0.856)

Scale from 1 = totally disagree to 5 = totally agree.

### 3.2. Students’ assessment of CHL and SL before and after the training course

Participation in the course was associated with a mean improvement of 0.12 points out of the total score of 5, but this is not significant (*p-*value = 0.05) (Table 4). A mean improvement of 0.04 points in SL and 0.13 Trust in Science was observed after the intervention, but these are not significant (*p-*value = 0.05) (Table 4). The average scores of students in the linguistic and scientific tracks for CHL, SL, and trust in science decreased between before and after training, −0.06, −0.13, and −0.12 respectively, but this difference is not significant. Students in the scientific track improved their scores for both CHL, SL, and trust in science as do those in the human science track; however, these differences are also not significant. The ANOVA test indicates that the difference in means among the specialization groups is significant concerning trust in science before training (*p-*value = 0.015). Students in the human science track are significantly different from those in the Linguistic and Scientific tracks, while students in the Scientific track do not differ from either the former or the latter group.

**Table 4:** Changes in CHL, SL, and trust in science scores with the training course.

	Baseline value	Post training change	*p*-value
CHL	3.08	0.12	0.171
Linguistic and scientific	3.08	−0.06	0.740
Scientific	3.13	0.31	0.84
Human science	3.05	0.15	0.223
SL	3.66	0.04	0.635
Linguistic and scientific	3.70	−0.13	0.458
Scientific	3.62	0.10	0.548
Human science	3.65	0.19	0.077
Trust in science	3.00	0.13	0.139
Linguistic and scientific	3.22	−0.12	0.306
Scientific	3.00	0.31	0.082
Human science	2.84	0.16	0.273

Scale from 1 = totally disagree to 5 = totally agree.

The first item of CHL (“When news arouses special emotions, I refrain from drawing immediate conclusions”) accounted for a significant change after intervention (*p-*value = 0.01). In contrast, the other items did not change significantly (*p-*value = 0.05). Items 5 (“I am able to define steps of scientific method”) and 10 (“I believe that before spreading information that seems revolutionary from the scientific point of view, it is important to deepen and ascertain its veracity through further research”) of the SL questionnaire resulted in a significant change after intervention (*p-*value = 0.02 and *p-*value = 0.032). The questionnaire assessing Trust in Science did not report any significant change on any item score.

Bivariate correlations (Table 5) suggested that individually Perceived Quality had a moderate to high positive significant correlation with Trust in Science (*p-*value 0.002; R 0.371), SL (*p-*value 0.000; R 0.510), and CHL scores after the training course (*p-*value 0.040; R 0.246). Trust in Science evaluated after intervention significantly correlated with SL (*p-*value 0.015; R 0.290) and CHL (*p-*value 0.005; R 0.331) scores after the course. CHL correlated significantly with SL scores (*p-*value 0.000; R 0.456) after the intervention.

**Table 5:** Correlation of primary and secondary outcomes post training.

	Trust in science (post)	SL (post)	CHL (post)	Perceived quality
Trust in science (post)		0.290*	0.331**	0.371*
SL (post)			0.456**	0.510**
CHL (post)				0.246*
Perceived quality				

**Correlation is significant at the 0.01 level (2-tailed).

*Correlation is significant at the 0.05 level (2-tailed).

### 3.3. The value of the course from students’ perspective

The post-questionnaire showed that most of the students were satisfied with the acquired skills (66%) and found them useful for everyday life (70%), close to real issues (75%), and essential competencies to be taught in school (65%). The average score in the Individually Perceived Quality questionnaire among students of all classes was 3.83 (total score: 5 points) with a St. deviation of 0.67, indicating general satisfaction among students. We were able to observe, however, that the perceived quality differed among the various specializations. Students in the linguistic and scientific track had an average score of 3.50, and the score increased to 3.84 for students in the scientific track and further to 4.07 for the human science track. The ANOVA test was conducted with a significance level of 0.012 (Table 6).

**Table 6:** Perceived quality mean score by specialization.

	Linguistic and scientific	Scientific	Human science
Mean (St. Dev)*	3.50 (0.7)	3.84 (0.7)	4.07 (0.5)

Scale from 1 = totally disagree to 5 = totally agree.

*Human science score is different from Linguistic and scientific, and ANOVA test is significant at the level 0.012.

The analysis of the open-ended questions highlighted the strengths, the limitations, and the room for improvement (Table 7).

**Table 7:** Themes identified through the questionnaire on perceived quality.

Positive aspects	Interaction	“the student is asked to answer questions about a concept just explained” “to intervene was highly encouraged”
	Clarity	“mastery of the technical language” “good speakers” “well exposed and understandable” “well structured”
	New concepts	“I learned to differentiate between scientific and non-scientific knowledge” “I learned several new things”
	Applicability	“applicable to daily contexts”
Negative aspects	Duration	“Short time, 4 h in a row are too much” “too long” “a little big boring”
	Theoretical	“I would add more practical exercise” “I would add more videos”
	Focus on the pandemic	“too many pandemic examples”
Aspects to be deepened	Critical thinking	“how to recognize the credibility of the source” “Disinformation and medical disinformation” “Manipulation” “Fake news”
	Scientific literacy	“scientific method” “the effect of pseudoscience in the society” “How to recognize conspiracy theories”
	Psychological aspects	“prejudices and stereotypes” “body language” “psychological aspects of critical thinking”

During the final meeting with the research team, the teachers provided further feedback: human science students reported an overall positive feeling and interest, especially in CHL themes, whereas scientific students, though generally satisfied, claimed to know most of the topics and their preference for a significant focus on SL during the training course. Students’ strong interest has been highlighted by their willingness to include the training topics in the transversal courses of the Baccalaureate examination.

## 4. Discussion

We developed and tested, for its feasibility and acceptability, a curriculum for secondary school students aimed at increasing CHL and SL in an institute in the Southern region of Switzerland. The curriculum proved to be feasible and well-accepted. Students convincingly specified their perceived personal benefits associated with a positive change in CHL and SL scores after the training. The increased perceived quality of the educational content was related to enhancing students’ learning rate. Quantitative results support the hypothesis that implementing the curriculum increased CHL and SL competencies.

The small positive increase in CHL and SL averages between the tests, even if not significant, may be partially explained by a generally positive disposition of students toward Critical Thinking assessed at the beginning of the course: this hypothesis is in line with previous studies reporting that an overall positive inclination toward Critical Thinking significantly contributes to the development of critical thinking skills (40). Students reported a significant change after the course in some items of the questionnaires assessing CHL and SL. The item “When news arouses special emotions, I refrain from drawing immediate conclusions” of the first questionnaire indicates a perceived enhancement in recognizing and reducing those emotions that often interfere with critical or rational thinking. This finding is consistent with previous research showing that, when we are emotional, our ability to think, decide and solve problems decreases (41–43). This notion is supported by recent neuroimaging results demonstrating that emotion-specific enhancement is associated with the suppression of brain activity within the valuation system (44) defined as a “brain framework for knowledge representation and inference” (45). This finding is highly relevant because it suggests that increasing the perceived ability to manage emotions enhances those abilities included in the broad framework of critical thinking. The significant score changes in the item “I am able to define steps of scientific method” of the questionnaire assessing SL indicate a perceived improvement in students’ ability for observation, formulation, testing hypothesis, measurement, and experiment.

Trust in Science positively correlates with SL and CHL. This confirms previous findings by Hendriks and his colleagues (46) that trust in science is crucial for SL. To engage with science-related issues, laypeople have primarily to trust in scientists and the scientific process. Contemporary, most scientific discoveries do not rely on firsthand experience, as observation and analysis of scientific phenomena are not always feasible without scientific instruments and competencies. For this reason, specialized knowledge is crucial to understanding scientific phenomena, making trust in science indispensable. That a strong positive relation exists between Trust in Science and CHL is relatively surprising and inconsistent with previous findings. Previous studies have found that highly educated people demonstrating more critical thinking tend to trust less science and scientists because they are more likely to search for information before deciding whom to trust (47). Yore et al. (48) reported that critical thinking is one of the elements of SL. When facing scientific information, scientifically literate individual judges what to consider or not based on those elements of critical thinking applicable in the scientific context. This shed light on the role of critical thinking in understanding science, decision-making, and problem-solving of scientific issues (49). Evaluating the credibility of scientific sources, assessing available evidence, recognizing the provision of science, and discerning science from pseudoscience, arise at the overlap of SL and CHL (18).

Our feasibility study also highlights a gap in the way students from different tracks perceive the course and its contents. Students from the linguistic and scientific tracks lowered their scores in relation to all primary outcomes, whereas those from the scientific track improved in all outcomes, along with those from the human science track. However, it should be noted that initial differences are significant only about trust in science, which is lower among students from the third track, the humanities track, compared to the other two. The impact of different tracks within upper secondary schools on students’ performance has recently been documented in a neighboring country. In this context, it was observed that students from classical tracks acquired more competencies (50). Similar differences are also noticeable in the assessment of the course quality. Although a modest judgment is present among students from the linguistic and scientific tracks, approval ratings increase among students from the scientific track and even more so among those from the humanities track. These results, triangulated with qualitative data and teacher feedback, underline the importance of targeting these types of curricula based on the specialization tracks.

Perceived quality also exhibited a positive correlation with all primary outcomes. This observation could imply that the students who derived the greatest benefit from the course were also the ones who held the highest level of appreciation for it. However, it could alternatively suggest that those participants who were already interested and competent were the ones who appreciated it the most. When addressing subjects related to science communication and public understanding of science, it is imperative to untangle these interpretations. Consequently, a large-scale study would be essential to independently examine the impact of the course and the predispositions of the participants.

### 4.1. Limitations

Our study has some limitations. It is a feasibility study conducted in one school with a convenience sample of students. For this reason, our ability to generalize findings is limited. The school accepting to pilot the intervention to improve CHL and SL presented a favorable attitude toward the topic, which may represent a selection bias increasing the chances of a successful outcome. For future research, we suggest conducting the course in more than one institute, including other high schools and technical schools, and structuring it into multiple shorter sessions covering different topics of interest in addition to pandemic ones. Another limitation refers to the measures used in our study. CHL and SL scales were not validated but self-constructed, and this may affect their validity and reliability. Indeed, reliability analysis yielded rather low scores. However, validated measures for the suggested operationalization of the two constructs are not yet available or they are far from the conceptual definition adopted by the authors of this paper. That the measurements are not validated may lower the ability to detect the effect of the course, even though this was not the main aim of the study. Another limitation is that the measures used were assessed participant self-perception and did not test an actual skills improvement. Large-scale studies should overcome both these limitations by developing and validating robust measurements.

## 5. Conclusion

Considering the propension of adolescents to develop critical thinking, improving CHL and SL should start in secondary schools. We report that training CHL and SL in secondary school students is feasible and can improve their competencies. The study highlighted the importance of building the didactic contents using a participatory approach to avoid overlaps with school curricula and, foremost, to understand specific gaps. A single didactic curriculum adapted to different secondary schools’ programs should be tested.

## Data availability statement

The raw data supporting the conclusions of this article will be made available by the authors, without undue reservation.

## Ethics statement

The studies involving humans were approved by Ethical Committee of the Università della Svizzera italiana. The studies were conducted in accordance with the local legislation and institutional requirements. Written informed consent for participation was not required from the participants or the participants’ legal guardians/next of kin because upon starting the online questionnaire, all participants were informed of the nature and aims of the study and that they could withdraw their consent to participate at any time. Informed consent was obtained at the start of the digital survey.

## Author contributions

MF, SR, and ND conceptualized the study and the paper, and structured its content. RF analyzed data and prepared the first draft of the manuscript after discussions with MF. MF finalized the first version of the manuscript and wrote the subsequent versions until the final. SR and ND provided feedback and suggestions for revision until consensus on the final version was reached. All authors contributed to the article and approved the submitted version.

## Funding

To support this study we received funding from the Swiss School of Public Health. Open access funding by Università della Svizzera italiana.

## Conflict of interest

The authors declare that the research was conducted in the absence of any commercial or financial relationships that could be construed as a potential conflict of interest.

## Publisher’s note

All claims expressed in this article are solely those of the authors and do not necessarily represent those of their affiliated organizations, or those of the publisher, the editors and the reviewers. Any product that may be evaluated in this article, or claim that may be made by its manufacturer, is not guaranteed or endorsed by the publisher.
