# Supplementary material for: Strengthening adolescents’ critical health literacy and scientific literacy to tackle mis- and dis-information. A feasibility study in Switzerland
Source: Front Public Health. 2023 Sep 1;11:1183838. doi: 10.3389/fpubh.2023.1183838 (PMC10505801; doi:10.3389/fpubh.2023.1183838)
Supplement: Supplementary file 1 [file Table_1.DOCX]

**Supplementary table 1. Critical Health Literacy scale score before-after the training**

|  | Mean (St.Dev)  pre-training | Mean (St.Dev)  post-training |
| --- | --- | --- |
| When a piece of news evokes emotions in me, I refrain from drawing immediate conclusions. | 3.11(0.9) | 3.63(0.9) |
| When I read a post on social media, I can assess whether the source is credible or not. | 3.43(1.0) | 3.71(0.9) |
| I believe that all opinions hold equal value, regardless of the competence of the individuals expressing them. | 2.50(1.2) | 2.31(1.1) |
| When I read news on social media, it is essential for me to verify its credibility. | 3.70(1.0) | 3.78(0.9) |
| In general, I rely on sources that share my views and avoid considering those that contradict my thoughts. | 2.41(0.9) | 2.31(0.9) |
| Mass media (TV, newspapers) and social media always provide an accurate portrayal of reality. | 1.85(0.8) | 2.10(0.9) |
| The physical appearance, gestures, likability, or tone of voice of a speaker are crucial factors for evaluating their credibility. | 2.79(1.3) | 2.49(0.9) |
| A few cases are enough to establish a general rule. | 2.04(0.8) | 2.01(0.9) |
| When I type a keyword on the Internet, the search results always display high-quality websites first. | 2.79(1.1) | 2.60(1.1) |
| Knowing a few people from a group is sufficient to understand the characteristics of all others belonging to the same group. | 2.00(0.9) | 2.14(1.1) |

*Source: Questionnaires administered to students at a secondary school in Switzerland before and after participation in a training on Critical Health Literacy and Scientific Literacy.*

*Base: 72 students.*

**Supplementary table 2. Scientific Literacy scale scores before-after the training**

|  | Mean (St.Dev)  pre-training | Mean (St.Dev)  post-training |
| --- | --- | --- |
| The results of science are never definitive but can change following other discoveries. | 4.29(0.6) | 4.08(1.0) |
| When a theory is confirmed, it remains valid forever. | 2.00(0.9) | 2.10(1.0) |
| Science is a technical knowledge, and therefore, discussing science requires expertise in the subject matter. | 3.99(0.8) | 3.86(0.9) |
| I can recognize a conspiracy theory. | 3.61(0.8) | 3.83(0.9) |
| I can describe the functioning of the scientific method. | 3.46(1.0) | 3.90(0.9) |
| Our senses and experiences are sufficient to understand things. | 2.50(0.8) | 2.35(0.9) |
| True science is based on observation and not on personal viewpoints. | 3.86(1.0) | 4.04(1.0) |
| I can recognize when a study is based on rigorous methods. | 3.35(0.8) | 3.49(0.8) |
| I can distinguish science from ideologies and superstitions. | 3.90(0.8) | 3.96(0.8) |
| I believe that before disseminating information that appears revolutionary from a scientific perspective, it is important to delve deeper and verify its accuracy through further research. | 4.46(0.6) | 4.06(0.9) |

*Source: Questionnaires administered to students at a secondary school in Switzerland before and after participation in a training on Critical Health Literacy and Scientific Literacy.*

*Base: 72 students.*

**Supplementary table 3. Trust in science scale scores before-after the training**

|  | Mean (St.Dev)  pre-training | Mean (St.Dev)  post-training |
| --- | --- | --- |
| People trust scientists much more than they should. | 2.97(1.0) | 2.93(1.0) |
| A lot of scientific theories are completely wrong. | 2.51(0.7) | 2.49(0.9) |
| Our society places too much emphasis on science. | 2.80(1.0) | 2.79(1.1) |
| We should trust the work of scientists. | 3.61(0.8) | 3.80(0.8) |
| We can rely on science to find the answers that explain the natural world. | 3.84(0.8) | 3.99(0.8) |

*Source: Questionnaires administered to students at a secondary school in Switzerland before and after participation in a training on Critical Health Literacy and Scientific Literacy.*

*Base: 72 students.*
